# Neighbourhood characteristics and children’s oral health: a multilevel population-based cohort study

**DOI:** 10.1093/eurpub/ckab013

**Published:** 2021-02-24

**Authors:** Agatha W van Meijeren-van Lunteren, Joost Oude Groeniger, Eppo B Wolvius, Lea Kragt

**Affiliations:** 1 The Generation R Study Group, Erasmus University Medical Centre, Rotterdam, The Netherlands; 2 Department of Oral & Maxillofacial Surgery, Special Dental Care and Orthodontics, Erasmus University Medical Centre, Rotterdam, The Netherlands; 3 Department of Public Health, Erasmus University Medical Centre, Rotterdam, The Netherlands; 4 Department of Public Administration and Sociology, Erasmus University, Rotterdam, The Netherlands

## Abstract

**Background:**

To understand determinants of oral health inequalities, multilevel modelling is a useful manner to study contextual factors in relation to individual oral health. Several studies outside Europe have been performed so far, however, contextual variables used are diverse and results conflicting. Therefore, this study investigated whether neighbourhood level differences in oral health exist, and whether any of the neighbourhood characteristics used were associated with oral health.

**Methods:**

This study is embedded in The Generation R Study, a prospective cohort study conducted in The Netherlands. In total, 5 960 6-year-old children, representing 158 neighbourhoods in the area of Rotterdam, were included. Data on individual and neighbourhood characteristics were derived from questionnaires, and via open data resources. Caries was assessed via intraoral photographs, and defined as decayed, missing and filled teeth (dmft).

**Results:**

Differences between neighbourhoods explained 13.3% of the risk of getting severe caries, and 2% of the chance of visiting the dentist yearly. After adjustments for neighbourhood and individual characteristics, neighbourhood deprivation was significantly associated with severe dental caries (OR: 1.48, 95% CI: 1.02–2.15), and suggestive of a low odds of visiting the dentist yearly (OR: 0.81, 95% CI: 0.56–1.18).

**Conclusions:**

Childhood caries and use of dental services differs between neighbourhoods and living in a deprived neighbourhood is associated with increased dental caries and decreased yearly use of dental services. This highlights the importance of neighbourhoods for understanding differences in children’s oral health, and for targeted policies and interventions to improve the oral health of children living in deprived neighbourhoods.

## Introduction

Despite improvements in recent years, low-socioeconomic households are still affected by poor oral health and its negative consequences over the life-course.^1^ In The Netherlands, large inequalities in oral health and dental care use among children exist, despite the fact that dental care for children is fully covered by basic health insurance.^2^

Extant studies have mainly focussed on investigating the relationship between individual level determinants and oral health.^3^^,^^4^ However, these individual characteristics could not fully explain disparities in dental caries and dental care utilization, and the success of individual behaviour interventions to reduce oral health inequalities is limited so far.^5^^,^^6^ As a result, the interest to research contextual determinants of oral health, has increased in the last years.^4^^,^^5^^,^^7^ Especially the physical and social environment consist of important determinants that may contribute to inequalities in oral health.^7^^,^^8^ For example, it is widely known that an unhealthy diet, including frequent consumption of sugars, increases the risk of dental caries, and that the food choices individuals make is dependent on the availability of healthy foods in the area where they live.^9^ Also, early preventive care by dentists is important to reduce the risk of childhood dental caries. However, receiving dental care may be dependent on access to and availability of dental health services in the area where individuals live.^10^ Lastly, socioeconomic characteristics of the neighbourhood (e.g. the level of neighbourhood deprivation) may also be an important determinants for oral health outcomes.^4^^,^^8^ While there have been several studies that found an association between contextual socioeconomic circumstances and oral health, it is not always clear whether this reflects features of the area or individual characteristics of the residents within the area.^8^^,^^11^ Therefore, it is of importance to consider the socioeconomic circumstances of the individuals itself, as well as those of their neighbourhood, when studying the relationship between contextual factors and individual oral health outcomes.

An appropriate manner to study contextual factors in relation to individual health outcomes is multilevel modelling. Multilevel modelling enables researchers to simultaneously analyse the effects of both neighbourhood and individual level, and accounts for the dependency of individuals living in the same area.^12^ Few studies have used multilevel analyses to investigate the relation between contextual determinants and oral health among children.^13–17^ Two studies investigated whether the number of dentists in a neighbourhood was associated with dental care use and dental caries, but no associations were observed.^15^^,^^17^ A study in Japan showed that the number of grocery stores per resident was positively associated with dental caries.^17^ Antunes et al. observed that in Brazil an increased level of the human development index (a composite measure summarizing neighbourhood income, instructional attainment and longevity) was associated with a lower number of untreated carious lesions^13^, although this finding was not observed in another Brazilian study.^16^ Lastly, whereas two studies in Brazil and Japan found that a higher average income per neighbourhood was associated with decreased dental caries in children^14^^,^^17^, this association was not observed in another Brazilian study.^16^

Because results of previous studies examining contextual determinants of oral health are inconclusive, and studies in Europe have not yet adopted multilevel analyses, this research combines neighbourhood data with individually collected data from The Netherlands in a multilevel framework to study: (i) whether neighbourhood level differences in caries and dental service use exist, and (ii) whether supermarket availability, snack bar availability, dentist availability, and neighbourhood deprivation level are associated with dental caries and dental services use.

## Methods

**Figure 1.**
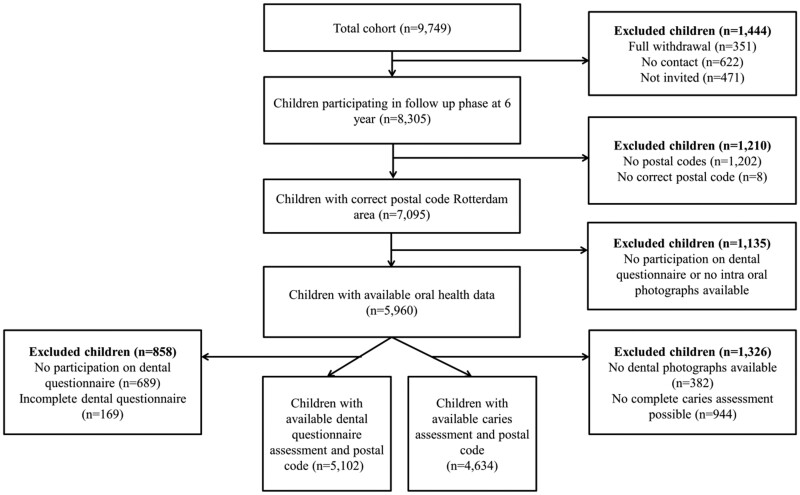
Flowchart showing the selection of the study population

This study is embedded in The Generation R Study, a population-based prospective cohort study from foetal life onwards conducted in Rotterdam, The Netherlands.^18^ All pregnant mothers living in Rotterdam expecting to deliver between April 2002 and January 2006 were invited to participate. Data collection started during pregnancy, was continued prenatally, and is still ongoing at various time points through several data collection methods.^18^ For the current study, all data were collected when the children were 6 years. In this phase 8,305 (85% of original cohort (*n* = 9 749) children participated in the study, of which 5 960 children were eligible for this study (Figure 1). The study was approved by the Medical Ethical Committee of Erasmus Medical Centre, Rotterdam, The Netherlands (MEC 198.782/2001/31) and conducted according to the World Medical Association Declaration of Helsinki. Written informed consent was obtained from all participants. Water supplies were not fluoridated during the study period in Rotterdam.

### Neighbourhood characteristics

The following contextual factors on neighbourhood level were studied: supermarket availability, snack bar availability, dentist availability, and deprivation level. In The Netherlands, communities consist of districts, and districts are subdivided into neighbourhoods, which is determined by Statistics Netherlands. Moreover, the postal company in The Netherlands has subdivided each community in a set of postal codes, which almost correspond with the neighbourhood division.

The mean number of supermarkets and snack-bars within 1 km distance for all inhabitants living per neighbourhood in the year 2010, were available as open source data by Statistics Netherlands.^19^ For a postal code area that corresponded with more than one neighbourhood, the mean of the neighbourhood variables of areas with similar postal code was calculated.

Dental clinic availability was retrieved via a registry, managed by Vektis, that contains all health care providers and their working locations in The Netherlands per postal code for the year 2010.^20^ For the analyses, the number of dental clinics per 10 000 inhabitants was calculated and used.

Neighbourhood deprivation was determined by neighbourhood status scores (NSSs) of the year 2010 derived from The Netherlands Institute for Social Research.^21^ These scores are calculated for all postal codes in The Netherlands on the basis of four characteristics: average income, unemployed residents, residents with low education and households with low income. Analyses were performed using a categorical scale of the NSS: low NSS (< −1), middle NSS (−1–1), high NSS (>1), which is based on the standard deviation of the NSS in The Netherlands.^21^

### Oral health outcomes

For this study two outcomes were analysed: dental caries and dental care use.

From October 2008–January 2012, 5 578 children visited the research centre for hands-on measurements. After tooth brushing, 10 photographs of clean teeth were taken using an intra-oral camera (Poscam USB intra-oral autofocus camera, Digital Leader PointNix, 640 × 480 pixels). All photographs were scored by one single calibrated dentist, and 10% of the photographs were scored by a second dentist using the same method. Intrarater-reliability (Cohen’s kappa = 0.80) and inter-observer reliability (Cohen’s kappa = 0.76) were evaluated and both showed good agreement.^22^ Dental caries was assessed in the primary dentition using the decayed, missing, and filled teeth (dmft) index.^23^ Decayed teeth were assessed as lesions extended into dentin, enamel caries was not taken into account. Missing teeth were only assessed when teeth were extracted due to caries, which was individually judged based on the dental development and caries pattern of the child. Filled teeth were scored if teeth were restored due to caries. The use of intra oral photographs for scoring dmft in epidemiological studies showed high sensitivity and specificity (85.5% and 83.6%, respectively) compared to the clinical visual tactile inspection.^22^

Dental visits were assessed by means of parental questionnaires, in which parents answered the question whether their child had visited the dentist in the past year (yes/no).

### Covariates

Socioeconomic status (SES) was retrieved via parental questionnaires and measured using: maternal education level, net household income, maternal employment status, and marital status. Educational level was defined as: low (no education, primary education, ≤ 4 years general secondary school or lower vocational training), middle (>4 years general secondary school or intermediate vocational school), and high (bachelor’s degree, higher vocational school or a university degree finished).^24^ Monthly net household income was categorized as ‘≤ €2400’ and ‘> € 2400’, based on the average monthly general labour income in The Netherlands in 2010.^25^ Employment status of the mother was dichotomized as ‘paid job’ or ‘no paid job’. Marital status of the mother was dichotomized as married (married or registered partnership) or not. Children’s ethnic background was defined according to the Dutch classification of ethnic background and classified as ‘Dutch’ if both parents of the child were born in The Netherlands and ‘non-Dutch’ if one of the parents was born in another country than The Netherlands.^26^ Sugar intake during childhood was assessed in questionnaires with questions about the frequency of consuming high caloric snacks and sugar containing beverages. For the analyses, sugar intake was dichotomized as ‘low’ (≤2 sugar containing products a day) and ‘high’ (≥3 sugar containing products a day). Tooth brushing frequency was assessed by means of questionnaires and dichotomized as ‘≤1 per day’, or ‘≥2 per day’.

### Data analyses

Multilevel logistic regression models were used to estimate Odds Ratios (ORs) of having mild (dmft 1–3) or severe caries (dmft > 3) compared to children with no caries (dmft = 0). Multilevel models are useful to study clustered data, as in this study where children (level-1) are clustered within neighbourhoods (level-2). We used random intercept multilevel models for all analysis. In these models, the intercept is allowed to vary across neighbourhoods thereby accounting for the clustering of children within neighbourhoods. We verified that the relationship between each continuous predictor and the outcome was linear on the logit scale, and that multicollinearity between predictor variables was absent. We constructed three models for each dental outcome:

1. Null model: this is an empty model which enabled to observe the proportion of the total variance that is due to neighbourhood differences. The variance partitioning coefficient (VPC) was calculated using a method where the individual level variance is fixed at 3.29 (π2/3) for dichotomous outcome variables.^27^ The percentage neighbourhood variance was calculated by dividing the random intercept variance (neighbourhood level variance component) by the sum of the individual and neighbourhood level variances. The VPC can vary between 0 and 100%, the higher this percentage the larger the role of neighbourhoods in the existing difference of caries experience between individuals. The VPC was calculated per imputed dataset and consequently averaged to present one summary VPC per model.
2. Model 1: this model includes one of the four neighbourhood variables separately
3. Model 2: this model includes all neighbourhood variables simultaneously
4. Model 3: Model 2+the individual variables that were considered as confounders

Multiple imputation was performed to account for information bias associated with missing data in the covariates. Missing values were multiple imputed by generating 10 independent datasets with the use of chained equations, and effect estimates for each imputed dataset were pooled and presented in this study. Imputations were based on all variables in the models, but the main determinants and the outcomes were not imputed. Statistical analyses were generated using R 3.6.1 (R Core Team, Vienna, Austria) (packages: *mice* and *Lme4*). *P*-values ≤0.05 indicated statistical significance.

### Supplemental analyses

For the association between neighbourhood characteristics and dental caries, sugar consumption and brushing frequency were considered as mediators rather than confounding factors. To observe the influence of these variables on the effect estimates, we performed sensitivity analyses to additionally adjust our models (Supplementary tables S1 and S2). The same applies for dental caries as a potential mediator in the association between neighbourhood characteristics and dental visits (Supplementary table S3). A non-response analysis was conducted to evaluate potential selection bias by comparing the sample characteristics of children with (included) and without (excluded) available information on postal code and oral health outcomes (Supplementary table S4).

## Results

### Population characteristics

The prevalence of mild and severe caries in our study population was 19.6 and 13.4%, respectively. In the total study population 92.4% visited the dentist yearly. Children with severe caries lived in neighbourhoods with an average of 3.5 (±SD 2.1) supermarkets and 15.1 (±SD 14.0) snack bars which is higher than children without caries (mean ± SD 2.6 ± 2.0; and 10.0 ± 12.3, respectively). In addition, 54.5% of children with severe caries and 41.6% of children with mild caries lived in deprived neighbourhoods, compared with 30.7% of children without caries (Table 1).

**Table 1:** Individual and neighbourhood characteristics of the study population

Individual characteristics	Total population (*n = *5960)	No caries (*n = *3105)	Mild caries (*n = *909)	Severe caries (*n = *620)
Child’s gender				
Boys	3 003 (50.4%)	1 548 (49.4%)	438 (48.2%)	331 (53.4%)
Girls	2 957 (49.6%)	1 557 (50.1%)	471 (51.8%)	289 (46.6%)
Child’s age at dental assessment (mean ± SD)	6.2 ± 0.5	6.1 ± 0.4	6.3 ± 0.6	6.3 ± 0.6
Child’s age filling out questionnaire (mean ± SD)	6.1 ± 0.5	6.0 ± 0.4	6.2 ± 0.6	6.2 ± 0.6
Maternal educational level				
Low	745 (14.4%)	259 (9.5%)	132 (18.4%)	139 (32.1%)
Middle	1 699 (32.8%)	844 (30.9%)	271 (37.7%)	180 (41.6%)
High	2 735 (52.8%)	1 627 (59.6%)	315 (43.9%)	114 (26.3%)
*Missings*	*781 (13.1%)*	*375 (12.1%)*	*191 (21.0%)*	*187 (30.2%)*
Net income per month				
Low (< €2400)	1 618 (33.2%)	722 (27.9%)	279 (41.3%)	244 (60.1%)
High (> €2400)	3 262 (66.8%)	1 864 (72.1%)	397 (58.7%)	162 (39.9%)
*Missings*	*1 080 (18.1%)*	*519 (16.7%)*	*233 (25.6%)*	*214 (34.5%)*
Employment status mother				
Paid job	3 673 (74.9%)	2 073 (79.9%)	465 (68.3%)	211 (52.9%)
No paid job	1 231 (25.1%)	521 (20.1%)	216 (31.7%)	188 (47.1%)
*Missings*	*1 056 (17.7%)*	*511 (16.5%)*	*228 (25.1%)*	*221 (35.6%)*
Marital status				
Married/registered partnership	3 478 (67.0%)	1 790 (65.9%)	497 (68.6%)	313 (70.8%)
Unmarried/no registered partnership	1 714 (33.0%)	925 (34.1%)	228 (31.4%)	129 (29.2%)
*Missings*	*768 (12.9%)*	*390 (12.6%)*	*184 (20.2%)*	*178 (28.7%)*
Ethnic background				
Dutch	3 257 (55.8%)	1 859 (61.0%)	406 (46.2%)	170 (29.0%)
Non-Dutch	2 581 (44.2%)	1 188 (39%)	473 (53.8%)	416 (71.0%)
*Missings*	*122 (2.0%)*	*58 (1.9%)*	*30 (3.3%)*	*34 (5.5%)*
Sugar intake				
Low (≤2 per day)	1 637 (32.5%)	886 (33.5%)	224 (32.4%)	123 (29.0%)
High (>2 per day)	3 394 (67.5%)	1 756 (66.5%)	467 (67.6%)	301 (71.0%)
*Missings*	*929 (15.6%)*	*463 (14.9%)*	*218 (24.0%)*	*196 (31.6%)*
Tooth brushing per day				
≤ Once	1 056 (20.8%)	501 (19.0%)	147 (21.4%)	111 (25.8%)
≥ Twice	4 018 (79.2%)	2 136 (81.0%)	541 (78.6%)	320 (74.2%)
*Missings*	*886 (14.9%)*	*468 (15.1%)*	*221 (24.3%)*	*189 (30.5%)*
Dental visit in past year				
No	386 (7.6%)	210 (7.9%)	48 (6.9%)	27 (6.2%)
Yes	4 716 (92.4%)	2 435 (92.1%)	649 (93.1%)	407 (93.8%)
*Missings*	*858 (14.4%)*	*460 (14.8%)*	*212 (23.3%)*	*186 (30.0%)*
**Neighbourhood characteristics**				
Mean number of supermarkets within 1 km distance ± SD	2.8 ± 2.0	2.6 ± 2.0	2.9 ± 2.1	3.5 ± 2.1
Mean number of snack bars within1 km distance ± SD	10.6 ± 12.5	10.0 ± 12.3	11.5 ± 12.5	15.1 ± 14.0
Mean number of dental practices ± SD	3.2 ± 2.4	3.3 ± 2.5	3.1 ± 2.4	2.9 ± 2.2
Mean dental practice density per 10.000 inhabitants ± SD	3.3 ± 2.8	3.43 ± 2.9	3.1 ± 2.7	2.9 ± 2.4
Level of deprivation (mean NSS ± SD)	−0.5 ± 1.6	−0.3 ± 1.6	−0.7 ± 1.6	−1.2 ± 1.5
Low NSS (most deprived)	2 090 (35.1%)	954 (30.7%)	378 (41.6%)	338 (54.5%)
Middle NSS	2 322 (39.0%)	1 247 (40.2%)	334 (36.7%)	212 (34.2%)
High NSS (least deprived)	1 548 (26.0%)	904 (29.1%)	197 (21.7%)	70 (11.3%)

Numbers are presented as absolute numbers for categorical variables or as mean (SD) for continuous variables. NSS, neighbourhood status score. Missing values are presented in italic type as absolute numbers and percentages.

### Association between neighbourhood characteristics and dental caries

Differences between neighbourhoods explained 2.7% and 13.3% of the variance in mild and severe dental caries of 6-year-old children, respectively (Table 2, null model). Of the neighbourhood characteristics added in model 1, the VPC reduced the most for severe caries when NSS was added to the model (VPC: 5.0%). After controlling for individual characteristics (model 3), the VPC was (almost) 0% for both mild and severe caries. A statistically significant association was observed between neighbourhoods with middle NSS and low NSS and severe caries compared to neighbourhoods with high NSS (Table 2, model 2).The associations remained after adjustments for individual characteristics, although not significantly for middle NSS with severe caries (Model 3: middle NSS: OR: 1.32, 95% CI: 0.96–1.81; low NSS: OR: 1.48, 95% CI: 1.02–2.15, Table 2).

**Table 2:** Association between neighbourhood and dental caries**a**

	Null model		Model 1		Model 2		Model 3	
	Mild caries (dmft 1-3)	Severe caries (dmft >3)	Mild caries	Severe caries	Mild caries	Severe caries	Mild caries	Severe caries
Neighbourhood variables								
Number of supermarkets within 1 km distance	NA	NA	1.08 (1.03–1.13)	1.21 (1.12–1.31)	1.02 (0.93–1.12)	0.98 (0.86–1.12)	1.05 (0.96–1.14)	1.00 (0.90–1.12)
Number of snack bars within 1 km distance	NA	NA	1.01 (1.00–1.02)	1.03 (1.02–1.04)	1.00 (0.93–1.12)	1.02 (1.00–1.04)	0.99 (0.98–1.01)	1.01 (0.99–1.02)
Dental practice density per 10.000 inhabitants	NA	NA	0.97 (0.93–1.00)	0.96 (0.90–1.02)	0.98 (0.95–1.01)	0.95 (0.90–1.00)	1.00 (0.97–1.04)	0.99 (0.95–1.03)
NSS^b^ (deprivation score)								
Low NSS (most deprived)	NA	NA	1.86 (1.49–2.33)	4.58 (3.09–6.78)	1.73 (1.32–2.26)	3.42 (2.22–5.27)	1.10 (0.85–1.44)	1.48 (1.02–2.15)
Middle NSS	NA	NA	1.27 (1.01–1.59)	2.25 (1.51–3.35)	1.23 (0.98–1.55)	2.00 (1.36–2.93)	1.01 (0.82–1.25)	1.32 (0.96–1.81)
Neighbourhood variance	0.09	0.51	^c^	^d^	0.01	0.13	0.00	0.01
VPC	2.7%	13.3%	^c^	^d^	0.4%	3.7%	0%	0.2%

NSS, neighbourhood status score; VPC, variance partitioning coefficient (representing the proportion of variance due to neighbourhood level differences).

a Having no caries was reference category for all models. All analyses were performed using multilevel logistic binomial regression models, and results are presented as odds ratios (OR) with corresponding 95% confidence interval (CI).

b high NSS (least deprived) was reference category.

c The neighbourhood variance and VPC per model, respectively: Supermarkets: 0.07, 2.0%; Snack bars: 0.07, 2.1%; Dental practice density: 0.07, 2.1%; NSS: 0.02, 0.5%.

d The neighbourhood variance and VPC per model, respectively: Supermarkets: 0.36, 9.7%; Snack bars: 0.37, 10.1%; Dental practice density: 0.47, 12.4%; NSS: 0.17, 5.0%.

Null model: empty model with random intercept only.

Model 1: random intercept model per neighbourhood characteristic separately.

Model 2: model 1 + all neighbourhood characteristics (number of supermarkets, number of snack bars within 1 km distance. dental practice density, NSS).

Model 3: model 2 + individual characteristics (gender, age, maternal educational level, family household income, maternal employment status, maternal marital status, and ethnic background).

### Association between neighbourhood characteristics and dental visit

Differences between neighbourhoods explained 2% of the variance in yearly dental visits of 6-year-old children (Table 3, null model). The neighbourhood variance was 0% after including neighbourhood characteristics (Table 3, model 2). Compared to neighbourhoods with a high NSS, living in a neighbourhood with a low NSS decreased the likelihood of visiting the dentist (Table 3, model 2). This association remained after additional adjustment for individual characteristics, but was no longer statistically significant (Model 3: OR: 0.81, 95% CI: 0.56–1.18, Table 3).

**Table 3:** Association between neighbourhood and dental visit**a**

	Null model	Model 1	Model 2	Model 3
Neighbourhood variables				
Number of supermarkets within 1 km distance	NA	0.87 (0.83–0.91)	0.90 (0.80–1.00)	0.90 (0.80–1.01)
Number of snack bars within 1 km distance	NA	0.98 (0.98–0.99)	1.00 (0.98–1.02)	1.00 (0.99–1.02)
Dental practice density per 10.000 inhabitants	NA	1.02 (0.97–1.06)	1.01 (0.97–1.05)	1.00 (0.96–1.04)
NSS^b^ (deprivation score)	NA			
Low NSS (most deprived)	NA	0.49 (0.37–0.65)	0.68 (0.48–0.97)	0.81 (0.56–1.18)
Middle NSS	NA	0.69 (0.52–0.91)	0.76 (0.57–1.03)	0.81 (0.60–1.10)
(Range) Neighbourhood variance	0.07	^c^	0.00	0.00
(Range) VPC	2%	^c^	0%	0%

NSS, neighbourhood status score; VPC, variance partitioning coefficient (representing the proportion of variance due to neighbourhood level differences).

a Children that did not visited the dentist in the past year were the reference category for all models.

All analyses were performed using multilevel logistic binomial regression models, and results are presented as odds ratios (OR) with corresponding 95% confidence interval (CI).

b high NSS (least deprived) was reference category.

c The neighbourhood variance and VPC per model, respectively: Supermarkets: 0.00, 0%; Snack bars: 0.00, 0%; Dental practice density: 0.07, 2.1%; NSS: 0.00, 0%. Null model: empty model with random intercept only.

Model 1: random intercept model including neighbourhood characteristics.

Model 2: model 1+ all neighbourhood characteristics (number of supermarkets, number of snack bars within 1 km distance. dental practice density, NSS).

Model 3: model 2 + individual characteristics (gender, age, maternal educational level, family household income, maternal employment status, maternal marital status, and ethnic background).

## Discussion

The results of this study show that neighbourhood level differences in caries and dental health service use exist, but that these neighbourhood differences disappear after controlling for neighbourhood level and individual level characteristics. Living in a deprived neighbourhood is positively associated with dental caries and suggestive of decreased dental visits, even after adjusting for several individual socioeconomic characteristics.

Several studies have investigated the relationship between neighbourhood deprivation and oral health. In line with our results, three studies found a relationship between deprived areas and caries^13^, while two others did not^16^^,^^31^^,^^32^. However, only three studies used multilevel analyses similar to our study.^13^^,^^16^^,^^28^ Moreover, merely one of these studies controlled for individual socioeconomic indicators, which makes it difficult to conclude whether the poor oral health outcomes found in deprived areas reflect the individual SES or the physical and social environment individuals live in.^16^^,^^33^ In our study we used NSS as a measure of neighbourhood deprivation which is based on four sociodemographic characteristics. However, many other measures exist and using these may lead to different results. For example, a multilevel-study in the UK using area deprivation scores based on overcrowding in households, male unemployment, proportion of low SES, and proportion of persons without a car, did not find an association between area deprivation and the number of sound teeth among adults.^31^ Similarly, in an Italian multilevel study no association was observed between a city deprivation index and DMFT in 12-year-old children.^32^ However, whereas the latter study used the deprivation level of an entire city, we were able to assess neighbourhood deprivation levels within a city and villages, which gives a better understanding on how small local areas can influence oral health of children. Studies investigating area characteristics and dental service use are scarce, one previous study in England observed an association between neighbourhood deprivation and the use of dental services in elderly, which is comparable with the non-significant trend we observed in children.^34^

Our results indicate that the proportion of variance in dental caries and dental visits due to neighbourhood level differences is mostly accounted for by the included individual and neighbourhood characteristics. Still, the same models also showed that compared to non-deprived neighbourhoods, living in a deprived neighbourhood is associated with severe dental caries and a lower likelihood of visiting the dentist. In fact, the relevance of neighbourhood deprivation for oral health among children could indicate that contextual factors do matter, but that the administrative boundaries used in our study to differentiate between neighbourhoods might not be the most relevant for explaining variation in oral health. Alternatively, the relevance of neighbourhood deprivation may also suggest a residual effect of SES on oral health. Although we were able to adjust for several individual socioeconomic indicators, it is possible that the association between neighbourhood deprivation and poor oral health reflects the individual socioeconomic circumstances of the population living in deprived neighbourhoods.^33^ In absence of detailed information at various (lower) aggregate levels, we are not able to favour one explanation over the other and therefore we elaborate on the potential mechanism behind deprived neighbourhoods and oral health in the following paragraph.

There are several pathways via which living in a deprived neighbourhood could affect oral health. First, neighbourhoods can influence health via their physical characteristics.^7^^,^^9^ In our study, univariate models indicated that the number of supermarkets was associated with both oral health outcomes, but these associations attenuated after adjustments for other neighbourhood variables (Tables 1 and 2). Thus, the relationship between number of supermarkets and oral health is attributable to a higher number of supermarkets and snack bars in more deprived neighbourhoods, similarly shown before in other studies.^35^^,^^36^ Second, several theories exist through which the social environment in neighbourhoods could affect individual health.^7^ For example, Diez Roux and Mair suggest that neighbourhood safety, social connectedness and local institutions may affect health and corresponding behaviours.^7^ Other theories note that individuals living in the same neighbourhoods adapt their behaviours according to how others in the same geographical and social area behave.^37^ Overtime, predominant behaviours in an area can become a collective habitude, making the relation between neighbourhoods and health status bi-directional.^7^^,^^38^ This implies that the unfavourable oral health outcomes found in deprived neighbourhoods could reflect oral health-related behaviours of their inhabitants. However, individual behaviours such as sugar intake and brushing frequency did not influence our results (Supplementary tables S1 and S2). Also, dental caries experience was not related to dental visits (Table 1, and Supplementary table S3).

The results of this study have to be seen in the light of some limitations. Neighbourhood characteristics in this study were based on aggregated data, and it is important to acknowledge that this could have led to imprecise neighbourhood level data, causing non-differential misclassification. Also, our study relies on the assumptions of strict area borders, however, inhabitants might reside on the border of two areas and live closer to another neighbourhood. Although this would apply to a small number of children in our study population it could have led to slightly biased results. Furthermore, we have a large underestimation of the number of children not visiting the dentist on a yearly basis when observing regional statistics. In Rotterdam the percentage of children that did not visit the dentist in 2010 is 37.5% (range: 28.9%–45.5%), whereas in our study we only found that 7.6% of the children in our study population did not visit the dentist in the past year.^39^ This might be caused by misreporting of the caregivers, but it could also represent the selection bias due to differential participation in our study. The non-response analysis showed that the majority of excluded participants had missing postal codes and missing caries data (Supplementary table S4). However, excluded participants with available postal code data but missing dental caries data, lived more often in deprived neighbourhoods. On the other hand, children with missing postal codes but available dental caries data had a lower percentage of severe caries than children included in this study. This may suggest that our study either over- or underestimated the association between neighbourhood deprivation and dental caries. Finally, dental caries was assessed using intraoral photographs which makes it difficult to differentiate between stages of caries develaryopment. Therefore, only dentin caries was assessed and enamel caries was not evaluated. Although we do not expect that the distribution of non-cavitated lesions differs substantially from the distribution of cavitated lesions among our study population, an underestimation of caries may have caused a potential underestimation of the observed effect estimates.

The major strength of the current study is the large multi-ethnic study population based on a longitudinal cohort. In addition, this is one of the first studies in Europe using multilevel analyses to investigate area level influences on oral health. Also, we were able to make use of a diverse set of individual characteristics which increases the internal validity of this study.

## Conclusion

We have shown that children living in deprived neighbourhoods are at risk of dental caries and having fewer dental visits. This research shows that targeted policies and interventions related to oral health and related behaviour are needed for children living in deprived neighbourhoods. Unfortunately, information on what type of interventions might be effective to reduce dental caries in children living in deprived neighbourhoods is limited. Research on other health outcomes suggests that influencing the larger socioeconomic circumstances could improve health, instead of medical oriented interventions.^40^ We encourage future research to understand which interventions reduce the dental caries risk of children living in deprived neighbourhoods efficiently in order to develop targeted oral health interventions that have the potential to specifically decrease dental caries among children within deprived neighbourhoods.

## Supplementary Material

ckab013_Supplementary_Data
