# Supplementary material for: Neighbourhood characteristics and children’s oral health: a multilevel population-based cohort study
Source: Eur J Public Health. 2021 Feb 24;31(4):742–8. doi: 10.1093/eurpub/ckab013 (PMC8514066; doi:10.1093/eurpub/ckab013)
Supplement: ckab013_Supplementary_Data [file ckab013_supplementary_data.docx]

***Supplementary material***

**Article title:** Neighbourhood characteristics and children’s oral health: a multilevel population-based cohort study
**Authors:** Agatha W. van Meijeren - van Lunteren, Joost Oude Groeniger, Eppo B. Wolvius, and Lea Kragt
**Content of this file:** This electronic supplementary material contains tables with the results of several supplementary analyses which support the results of the main manuscript.

Table S1 – Association between neighbourhood and dental caries, including brushing frequency as mediator^a^

|  | Mild caries (dmft 1-3) | Severe caries (dmft >3) |
| --- | --- | --- |
| Neighbourhood variables |  |  |
| Number of supermarkets within 1 km distance | 1.05 (0.96-1.14) | 1.00 (0.99-1.11) |
| Number of snack bars within 1 km distance | 0.99 (0.98-1.01) | 1.01 (0.99-1.02) |
| Dental practice density per 10.000 inhabitants | 1.00 (0.97-1.04) | 0.99 (0.95-1.03) |
| NSS^b^ (deprivation score) |  |  |
| Low NSS (most deprived) | 1.10 (0.84-1.43) | 1.47 (1.02-2.13) |
| Middle NSS | 1.01 (0.82-1.25) | 1.31 (0.95-1.03) |

NSS=neighbourhood status score

^a^ Having no caries was reference category for the models. Analyses were performed using multilevel logistic binomial regression models, and results are presented as odds ratios (OR) with corresponding 95% confidence interval (CI)

^b^ high NSS (least deprived) was reference category

models were adjusted for: neighbourhood characteristics (number of supermarkets, number of snack bars within 1 km distance, dental practice density and NSS) and individual characteristics (gender, age, maternal educational level, family household income, maternal employment status, maternal marital status, ethnic background and brushing frequency)

Table S2 – Association between neighbourhood and dental caries, including sugar intake as mediator^a^

|  | Mild caries (dmft 1-3) | Severe caries (dmft >3) |
| --- | --- | --- |
| Neighbourhood variables |  |  |
| Number of supermarkets bars within 1 km distance | 1.05 (0.96-1.15) | 1.00 (0.90-1.12) |
| Number of snack bars within 1 km distance | 0.99 (0.98-1.01) | 1.01 (0.99-1.02) |
| Dental practice density per 10.000 inhabitants | 1.00 (0.97-1.04) | 0.99 (0.95-1.03) |
| NSS^b^ (deprivation score) |  |  |
| Low NSS (most deprived) | 1.10 (0.84-1.43) | 1.48 (1.02-2.15) |
| Middle NSS | 1.01 (0.82-1.25) | 1.32 (0.96-1.81) |

NSS=neighbourhood status score

^a^ Having no caries was reference category for the models. Analyses were performed using multilevel logistic binomial regression models, and results are presented as odds ratios (OR) with corresponding 95% confidence interval (CI)

^b^ high NSS (least deprived) was reference category

All models were adjusted for: neighbourhood characteristics (number of supermarkets, number of snack bars within 1 km distance, dental practice density and NSS) and individual characteristics (gender, age, maternal educational level, family household income, maternal employment status, maternal marital status, ethnic background and sugar intake)

Table S3 - Association between neighbourhood and dentist visit, including dental decay as mediator ^a^

| Neighbourhood variables |  |
| --- | --- |
| Number of supermarkets within 1 km distance | 0.90 (0.80-1.01) |
| Number of snack bars within 1 km distance | 1.00 (0.99-1.02) |
| Dental practice density per 10.000 inhabitants | 1.00 (0.96-1.04) |
| NSS^b^ (deprivation score) |  |
| Low NSS (most deprived) | 0.80 (0.55-1.16) |
| Middle NSS | 0.81 (0.60-1.09) |

NSS=neighbourhood status score

^a^ Children that did not visited the dentist in the past year were the reference category. Analyses were performed using multilevel logistic binomial regression models, and results are presented as odds ratios (OR) with corresponding 95% confidence interval (CI)

^b^ high NSS (least deprived) was reference category

models were adjusted for: neighbourhood characteristics (number of supermarkets, number of snack bars within 1 km distance, dental practice density and NSS) and individual characteristics (gender, age, maternal educational level, family household income, maternal employment status, maternal marital status, ethnic background and dental decay)

**Table S4 - Non-response analysis comparing characteristics of the study population with the excluded population at follow-up at the age of 6**

|  | Included (n=5960) | Excluded (n=2345) |
| --- | --- | --- |
| **Individual characteristics** |  |  |
| Child’s gender, *n* (%) |  |  |
| Boys | 3003 (50.4) | 1186 (50.6) |
| *Missings, n (%)* | *-* | *2 (0.1)* |
| Child’s age |  |  |
| Mean (SD) | 6.2 (0.5) | 6.2 (0.6) |
| *Missings, n (%)* | *382 (6.4)* | *1233 (52.6)* |
| Maternal educational level, *n* (%) |  |  |
| Low | 745 (14.4) | 58 (5.4) |
| Middle | 1699 (32.8) | 214 (19.9) |
| High | 2735 (52.8) | 803 (74.7) |
| *Missings, n (%)* | *781 (13.1)* | *1270 (54.2)* |
| Net income per month, *n* (%) |  |  |
| Low (<2400) | 1618 (33.2) | 187 (18.4) |
| *Missings, n (%)* | *1080 (18.1)* | *1331 (56.8)* |
| Employment status mother, *n* (%) |  |  |
| Paid Job | 3673 (74.9) | 767 (75.6) |
| *Missings, n (%)* | *1056 (17.7)* | *1330 (56.7)* |
| Marital status, *n* (%) |  |  |
| Married/registered partnership | 3478 (67.0) | 757 (71.3) |
| *Missings, n (%)* | *768 (12.9)* | *1283 (54.7)* |
| Ethnic background, *n* (%) |  |  |
| Non-Dutch | 2581 (44.2) | 887 (41.6) |
| *Missings, n* (%) | *122 (2.0)* | *213 (9.1)* |
| Sugar intake, *n* (%) |  |  |
| High (>2 times per day) | 3394 (67.5) | 644 (62.6) |
| *Missings, n (%)* | *929 (15.6)* | *1317 (56.2)* |
| Tooth brushing per day, *n* (%) |  |  |
| Once or less | 1056 (20.8) | 213 (21.7) |
| *Missings, n (%)* | *886 (14.9)* | *1364 (58.2)* |
| **Neighbourhood characteristics** |  |  |
| Mean number of supermarkets within 1 km distance ± SD | 2.7 (2.0) | 3.1 (2.1) |
| *Missings, n (%)* | *-* | *1208 (51.5)* |
| Mean number of snack bars within1 km distance ± SD | 10.6 (12.5) | 12.7 (12.9) |
| *Missings, n (%)* | *-* | *1208 (51.5)* |
| Mean dental practice density per 10.000 inhabitants ± SD | 3.3 (2.8) | 2.8 (2.4) |
| *Missings, n (%)* | *-* | *1208 (51.5)* |
| Level of deprivation |  |  |
| Low NSS (most deprived) | 2090 (35.1) | 550 (48.5) |
| Middle NSS | 1548 (26.0) | 168 (14.8) |
| High NSS (least deprived) | 2322 (39.0) | 417 (36.7) |
| *Missings,* *n* (%) | *-* | *1210 (51.6)* |
| **Outcome of interest** |  |  |
| Caries experience at 6 yr, *n* (%) |  |  |
| No (dmft=0) | 3105 (67.0) | 544 (78.5) |
| Mild (dmft 1-3) | 909 (19.6) | 108 (15.6) |
| Severe (dmft>3) | 620 (13.4) | 41 (5.9) |
| *Missings, n (%)* | *1326 (22.2)* | *1652 (70.4)* |
| Dentist visit in past year, *n* (%) |  |  |
| No | 386 (7.6) | *64 (6.6)* |
| *Missings, n (%)* | *858 (14.4)* | *1373 (58.6)* |

Numbers are presented as absolute numbers for categorical variables or as mean (SD) for continuous variables. NSS=neighbourhood status score
